# Junin Virus Triggers Macrophage Activation and Modulates Polarization According to Viral Strain Pathogenicity

**DOI:** 10.3389/fimmu.2019.02499

**Published:** 2019-10-22

**Authors:** María F. Ferrer, Pablo Thomas, Aída O. López Ortiz, Andrea E. Errasti, Nancy Charo, Victor Romanowski, Juan Gorgojo, María E. Rodriguez, Eugenio A. Carrera Silva, Ricardo M. Gómez

**Affiliations:** ^1^Laboratorio de Virus Animales, Instituto de Biotecnología y Biología Molecular, CONICET-Universidad Nacional de La Plata, La Plata, Argentina; ^2^Laboratorio de Trombosis Experimental, Instituto de Medicina Experimental, CONICET-Academia Nacional de Medicina, Buenos Aires, Argentina; ^3^Facultad de Medicina, Instituto de Farmacologia, University of Buenos Aries, Buenos Aires, Argentina; ^4^Global Viral Network, Baltimore, MD, United States; ^5^Centro de Investigación y Desarrollo en Fermentaciones Industriales, CONICET-Universidad Nacional de La Plata, La Plata, Argentina

**Keywords:** junin virus, human macrophages, TAM receptors, macrophage activation, macrophage polarization, IFN-I

## Abstract

The New World arenavirus Junin (JUNV) is the etiological agent of Argentine hemorrhagic fever (AHF). Previous studies of human macrophage infection by the Old-World arenaviruses Mopeia and Lassa showed that while the non-pathogenic Mopeia virus replicates and activates human macrophages, the pathogenic Lassa virus replicates but fails to activate human macrophages. Less is known in regard to the impact of New World arenavirus infection on the human macrophage immune response. Macrophage activation is critical for controlling infections but could also be usurped favoring immune evasion. Therefore, it is crucial to understand how the JUNV infection modulates macrophage plasticity to clarify its role in AHF pathogenesis. With this aim in mind, we compared infection with the attenuated Candid 1 (C#1) or the pathogenic P strains of the JUNV virus in human macrophage cultures. The results showed that both JUNV strains similarly replicated and induced morphological changes as early as 1 day post-infection. However, both strains differentially induced the expression of CD71, the receptor for cell entry, the activation and maturation molecules CD80, CD86, and HLA-DR and selectively modulated cytokine production. Higher levels of TNF-α, IL-10, and IL-12 were detected with C#1 strain, while the P strain induced only higher levels of IL-6. We also found that C#1 strain infection skewed macrophage polarization to M1, whereas the P strain shifted the response to an M2 phenotype. Interestingly, the MERTK receptor, that negatively regulates the immune response, was down-regulated by C#1 strain and up-regulated by P strain infection. Similarly, the target genes of MERTK activation, the cytokine suppressors SOCS1 and SOCS3, were also increased after P strain infection, in addition to IRF-1, that regulates type I IFN levels, which were higher with C#1 compared with P strain infection. Together, this differential activation/polarization pattern of macrophages elicited by P strain suggests a more evasive immune response and may have important implications in the pathogenesis of AHF and underpinning the development of new potential therapeutic strategies.

## Introduction

Junin virus (JUNV) is the etiological agent of Argentine hemorrhagic fever (AHF), an endemoepidemic disease mainly affecting agricultural workers in Argentina. The infection is usually acquired through small abrasions in the skin or through aspiration of particles contaminated with urine, saliva, or blood from carrier rodents. The AHF incubation period ranges from 6 to 12 days, ending with the onset of fever. During first 7 days, patients are commonly associated with a flu-like syndrome and the fever persists until the second week, when hemorrhagic or neurological signs of varied severity may be present. The 80% of patients improve after the second week. AHF diagnosis is based on clinical and laboratory data, the latter mainly based on platelet counts below 100,000/mm^3^ in combination with white blood cell counts under 2,500/mm^3^. Early diagnosis is important, because the early use of immune plasma from convalescent patients reduces mortality rates from 20 to 1%. Candid #1 (C#1) is a live attenuated vaccine against AHF, which is licensed in Argentina and has been administered to several hundred thousand persons in endemic areas for more than 20 years, with a major impact on the incidence of the disease. However, since the first description of the disease in the 1950s, uninterrupted annual outbreaks have been observed in a progressively expanding region in north-central Argentina, to the point that more than 5 million individuals are today considered to be at risk of AHF (1).

JUNV belongs to the clade B New World (NW) of genus mammarenavirus that together with genus reptarenavirus form the *Arenaviridae* family (2). Most mammarenavirus are associated with rodent infections. The Old World (OW) choriomeningitis lymphocytic virus (LCMV) infects *Mus musculus*, and this explains its global distribution. In contrast, other strains of mammarenavirus infect rodents with a circumscribed geographical distribution that explains their endemicity (3). In their natural rodent host, mammarenavirus usually produce a persistent asymptomatic infection that may occasionally be transmitted to humans where it can cause severe hemorrhagic fever (HF). In addition to JUNV, other strains of mammarenavirus associated with HF are the NW Machupo (MACV) and Chapare (CHPV) viruses in Bolivia, Sabiá (SABV) in Brazil and the OW Lassa virus (LASV) in Africa. In contrast, other members such as the NW Tacaribe (TCRV) and Pichindé (PICV) or the OW Mopeia (MOPV) viruses do not cause disease (4). The mammarenavirus are etiological agents of emergent diseases because human activity facilitates contact with wild rodents in new ecological niches and, therefore, new isolates should be expected in the future (3).

Like other members of the same family, JUNVs are enveloped virions, ~120 nm in diameter, with a capsid of helicoidal symmetry that includes a variable number of ribosomes. The virions contain a bi-segmented single-stranded RNA genome, with both segments employing an ambisense coding strategy. The L segment contains genes encoding the RNA-dependent RNA polymerase (L) and the matrix protein (Z). However, the smaller S segment encodes the nucleoprotein (N) and the glycoprotein precursor (GPC) which, after post-translational cleavage, yields mature virion glycoproteins (G) G1, G2 and the stable signal peptide SSP that together will constitute the spikes that decorate the virus surface (5).

Macrophages are the most functionally diverse (plastic) cells of the hematopoietic system. Macrophages are found in all tissues and their main function is to respond to pathogens and modulate the adaptive immune response through antigen processing and presentation (6, 7). Macrophage activation has emerged as a key area of study in immunology, tissue homeostasis, disease pathogenesis and inflammation resolution (8). To accomplish such diverse functions, they mature under the influence of signals from the local microenvironment into either classical M1 or alternatively M2 activated macrophages. M1 macrophages are characterized by the production of high levels of pro-inflammatory cytokines, an ability to mediate resistance to pathogens, strong microbicidal properties, high production of reactive nitrogen and oxygen intermediates and promotion of Th1 responses. In contrast, M2 activated macrophages are characterized by their involvement in parasite control, resolution of inflammation, tissue remodeling, immune regulation, and Th2 promotion responses (6, 9).

In this study, we aimed to characterize the infection of macrophages using two strains of the same arenavirus with different pathogenic properties. For this purpose, we studied the infection of human macrophages by the attenuated C#1 and the pathogenic P strains of JUNV, using an *in vitro* model represented by human macrophage cell cultures.

## Materials and Methods

### Cells

BHK-21 and Vero-76 cells (ATCC, USA) were maintained as monolayers, as previously described (10). Peripheral blood mononuclear cells (PBMCs) were obtained from healthy volunteer donors who had not taken any non-steroidal anti-inflammatory drugs for 10 days prior to sampling as previously described (11). This study was approved by the Institutional Ethics Committee, National Academy of Medicine, Argentina. Written consent was obtained from all subjects. Human monocyte-derived macrophages (HMDM) were obtained as previously reported (12). Briefly, PBMCs from healthy donors were isolated by Ficoll-Hypaque (GE, Chicago, IL, USA) density gradient centrifugation, and positive selection of CD14^+^ monocytes was performed using an EasySep™ Human CD14 Positive Selection Kit (StemCell Tech, Vancouver, Canada). Macrophage differentiation was carried out by plating 2.5 × 10^5^ CD14^+^ monocytes in 48-well plates containing 500 μL of RPMI 1640 plus 10% Fetal Bovine Serum (FBS) and 1% penicillin/streptomycin (PS) in the presence of rM-CSF (40 ng/ml) and cultured for 7 days. In selected experiments, 24-well plates were used with a double quantity of cells and medium.

### Virus

A virulent strain of JUNV, originally isolated from an AHF patient (P3441), as well as the attenuated Candid 1 (C#1) have been already described (13). The preparation of viral stocks in BHK-21 cells and infectivity titration using the Vero-76 cell line has been previously described (13). All work with the infective P strain was performed in a BSL/3 facility by vaccinated personal.

### Reagents

MEM, RPMI, and FBS were purchased from Invitrogen (Buenos Aires, Argentina). rM-CSF was acquired from R&D Systems (Minneapolis, MN, USA). Anti JUNV antibodies were obtained from BEI resources, USA. Anti CD71, CD14, CD86, CD80, HLA-DR, CD11b, CD11c, CD64, CD163, CD206 were obtained from BioLegend (San Diego, CA, USA). Anti-human APC-MERTK (mouse IgG1), Biotin-AXL (goat IgG) and isotype controls were obtained from R&D Systems (Minneapolis, MN, USA). Anti-TYRO3 (rabbit IgG) was obtained from Novus Biological (Littleton, CO, USA). DAPI was purchased from Invitrogen (Buenos Aires, Argentina). ELISA kits (Ready-SET-Go kits) for TNF-α, IL-1β, IL-6, IL-10, and IL-12p70 were obtained from eBioscience, Fisher scientific, Pittsburgh, PA, USA. Cytofix/Cytoperm kit was purchased from BD Bioscience (San Diego, CA, USA).

### Cell Infection

For viral infection, cells were washed with PBS twice before incubating with the virus at a multiplicity of infection (MOI) of 1 in serum free medium. After 1 h of incubation at 37°C, cells were washed with PBS twice again and supplemented with a complete culture medium. Mock infection was performed by adding the same volume of BHK-21 cell culture supernatant, instead of JUNV, to the cell monolayer. Cells were observed daily using an inverted microscope with an Olympus SP-320 camera and images were further processed with Photoshop 6.0 software.

### Plaque Formation Assay

Ten-fold dilutions of the macrophage-JUNV infected culture supernatants were added to 24-well plates with a 40–50% confluence monolayer of Vero E6 cells. The plate was then incubated at 37°C for 1 h with gentle rocking. Following adsorption, the inoculum was removed and overlaid with 2 ml of MEM containing 0.8% methylcellulose and 2% FBS and further incubated at 37°C in a humid atmosphere with 5% CO_2_. Plaques were allowed to develop for either 4–6 days before being fixed (4% w/v paraformaldehyde) and stained with a 1% Crystal Violet in 20% ethanol and _d_H_2_O.

### Indirect Immunofluorescence Studies

Cells were cultured on 12 mm diameter glass inserts before viral infection. At the indicated time-point after infection, the inserts were fixed with 4% paraformaldehyde (PFA) for 20 min and permeabilized with 0.1% Tween for 10 min. The slides were incubated overnight at 4°C with a pool of specific monoclonal antibodies against JUNV (13). FITC-conjugated anti mouse Igs were then applied to the PBS-washed slides for 30 min at room temperature (RT). Antibodies were diluted with PBS containing 5% FBS and 5% goat serum as blocking reagents. The slides were counterstained with DAPI and examined under a Nikon E200 microscope equipped with fluorescence filters and a 100-W mercury lamp. Images were acquired with a Tucsen TCC 5.0 refrigerated camera under the control of IS listen software and further processed using Photoshop 6.0 software.

### Flow Cytometry Analysis

The viability assay on macrophages culture was performed after 72 h of JUNV infection using Annexin V (AnnV) (Immunotools, Gladiolenweg, Friesoythe, Gemany) together with Fixable viability dye (eBioscience, USA). Briefly, cell harvesting was performed by a 20-min incubation with PBS plus 2% FBS (PBSF) and 1 mM EDTA on ice, followed by up and down pipetting. The harvested cells were washed once with PBSF and then stained with fixable viability dye efluor 780 diluted in PBS for 30 min. After washing cells, they were stained with AnnV following manufacturer's instruction. After final washing, the cells were fixed with a Cytofix/Cytoperm Kit (BD Biosciences, USA).

The surface staining for CD11b, CD64, CD206, CD14 (phenotypic characterization of macrophages) or CD11b, HLA-DR, CD86, and CD80 (activation status) was performed following a standard protocol. Briefly, the harvested cells were washed with PBS and blocked in PBSF on ice for 30 min. The cells were washed with PBS and the respective antibody cocktails (prepared in PBSF) were added to the cell pellet and incubated for 30 min on ice. A fixable viability dye was used according to the manufacturer's instructions to gate on live cells. After washing, the cells were fixed with a Cytofix/Cytoperm Kit, washed again and analyzed in a FACS Canto I (Becton Dickinson, Franklin Lakes, NJ, USA) or Partec-Sysmex CyFlow flow cytometer (Görlitz, Germany). All analysis was carried out with FlowJo software (Tree Star). Intracellular staining was performed following manufacturer's recommendation for the Cytofix/Cytoperm Kit. The preparation of blockage and cocktail antibodies was performed with PBSF. We have used fluorescent minus one (FMO) to set the threshold for each marker.

### Enzyme Linked Immunosorbent Assay (ELISA)

IL-6, IL-12p70, TNF-α, and IL-10 levels were assessed in culture supernatants with ELISA Ready-SET-Go kits (e-Bioscience) according to the manufacturer's protocol.

### RNA Isolation, RT-PCR, and Real-Time PCR

For gene expression analysis, cultured cells were washed and then harvested with Trizol (Life Technologies, Carlsbad, CA, USA) and total RNA was obtained following the manufacturer's instructions. Reverse transcription was performed using 100 ng of RNA by employing an iScript cDNA synthesis kit (Bio-Rad, Hercules, CA, USA). qPCR reactions were assessed using 1 μl of cDNA and using Sso Advanced Universal mix with Sybr Green and CFX-Connect equipment (Bio-Rad). Primers used in this study are listed in Table S1. The reaction was normalized to housekeeping gene expression levels and the specificity of the amplified products was checked through analysis of dissociation curves.

### Statistical Analysis

Each experiment was performed with 3–7 different donors. All results are graphed as the median (min-max, horizontal line indicates the median) and non-parametric one-way analysis of variance (ANOVA) (Kruskal–Wallis) followed by Dunn's multiple comparison test was used to detect significant differences between groups. In all cases, *P*-values lower than 0.05 were considered statistically significant. All statistical analyses were performed using Prism 6 software (GraphPad).

## Results

### JUNV Strains Replicate Similarly in Human Macrophages

**Figure 1:**
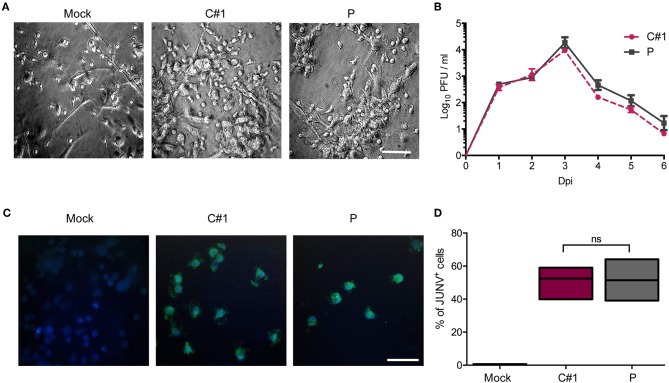
Human macrophages are equally susceptible to both JUNV variants. Human monocyte-derived macrophages (HMDM) cells were infected at a multiplicity of infection (MOI = 1) with the attenuated C#1 or the pathogenic P strains of JUNV. **(A)** Morphological observation of the cells. JUNV-infected cells become more flattened and extended. **(B)** Infective titration of cell supernatants was performed in Vero cells for 6 days by a standard PFU method. **(C)** Viral antigens were detected by immunofluorescence with a pool of antibodies and anti-JUNV-FITC, and nuclei were counterstained with DAPI. **(D)** HMDM cells were infected with C#1 or P strains and at 3 dpi cells were stained with anti-JUNV-FITC and the percentage of viral antigen positive cells were analyzed by flow cytometry. The percentage of infected cells was compared using the Non-parametric One-way ANOVA followed by Dunn's multiple comparison test. The results are graphed as the median (min-max, horizontal line indicates the median). At least three independent donors were used in each assay.

Human monocyte-derived macrophages (HMDM) cells were infected at a multiplicity of infection (MOI) = 1 with the attenuated C#1 or the pathogenic P strains of JUNV. HMDM cells showed clear morphological changes, such as becoming more flattened and extended, as early as 24 h post-infection (hpi) with both JUNV strains (Figure 1A). Infectious virus released to the cell culture supernatants were measured over 6 days by plaque formation assay. Infection with both viral strains led to similar levels of infectious viruses, peaking at 3 days post-infection (dpi) and declining until the end of the study (Figure 1B). Viral antigen was studied at 3 dpi by immunofluorescence (IF) and flow cytometry (FC) analysis. Viral protein staining was similarly positive with both strains (Figure 1C). As expected, FC analysis confirmed these results showing that 58% of HMDM cells were infected with C#1 strain meanwhile 57.6% were positive for the P strain (Figure 1D).

### JUNV Strains Differentially Enhances the Expression of CD71

**Figure 2:**
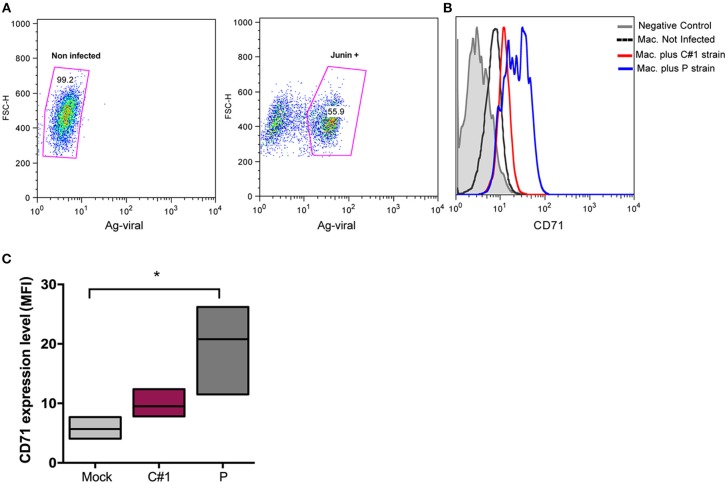
JUNV enhances CD71 expression in human macrophages with P showing the highest values. HMDM cells were infected at a multiplicity of infection (MOI = 1) with C#1 or P strains of JUNV. After 3 dpi, cells were double stained with anti-JUNV and anti-CD71 and analyzed by flow cytometry. **(A)** Representative dot-plots showing positive signal for viral antigen in macrophages. **(B)** Representative histogram showing CD71 expression after gating in positive cells for JUNV antigen and negative controls. **(C)** Expression level of CD71 analyzed as mean fluorescent intensity (MFI) in graph. Non-parametric One-way ANOVA followed by Dunn's multiple comparison test was used to detect significant differences between groups; *P < 0.05. The results are graphed as the median (min-max, horizontal line indicates the median) of at least four independent donors in each assay.

Viruses exploit fundamental cellular processes to gain entry into cells and deliver their genetic cargo. Virus entry pathways are largely defined by the interactions between virus particles and their receptors at the cell surface. These interactions determine the mechanisms of virus attachment and, ultimately, penetration into the cytosol. In contrast to LASV and other OW arenaviruses, which use α-dystroglycan to infect cells, the NW arenaviruses, including JUNV, use human transferrin receptor 1 (hTFR1 or CD71) (14). We have previously shown that JUNV infection enhances the expression of hTFR1 in the precursor CD34^+^ cells, suggesting that JUNV infection promotes its own dissemination (15). Compared with other cell types, mature macrophages may be atypical regarding the requirements for hTFR1 expression levels (16), and for that reason, we explored the expression pattern in HMDM infected cells. In this sense, our results showed that JUNV infection enhances CD71 expression in human macrophages, but with the highest value associated with P strain infection (Figures 2A–C).

### JUNV Strains Differentially Activate Macrophages and Cytokine Production

**Figure 3:**
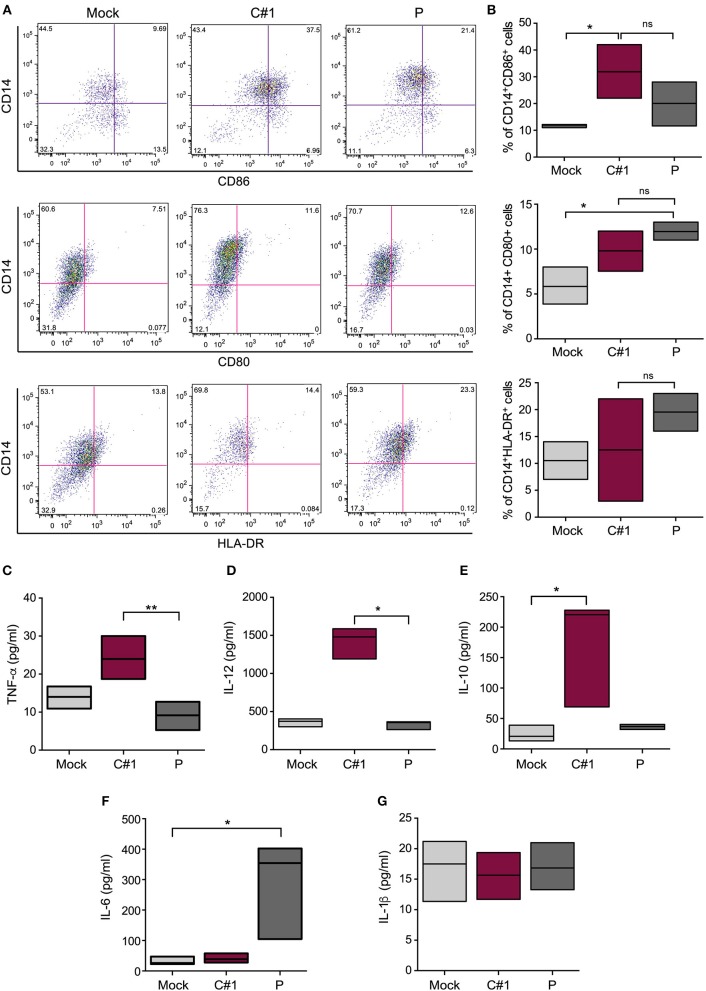
JUNV activates human macrophages and selectively modulates cytokine production. HMDM cells were infected with C#1 or P strains of JUNV (MOI = 1) and at 3 dpi the activation and co-stimulation CD80, CD86, or antigen presentation HLA-DR expression were evaluated in CD14^+^ cells by flow cytometry. **(A)** Representative dot-plots showing CD14^+^ and each marker are shown. **(B)** The percentage of double positive CD14^+^CD86^+^, CD14^+^CD80^+^, and CD14^+^HLA-DR^++^ are graphed. We had set the threshold for each marker based on the FMO. The expression level of TNF-α and IL-1β **(C)**, IL-12 **(D)**, IL-10 **(E)**, IL-6 **(F)**, and IL-1β **(G)** were measured in the supernatant of infected macrophages after 72 h, employing commercial ELISA kits. Non-parametric One-way ANOVA followed by Dunn's multiple comparison test was used to detect significant differences between groups, *P < 0.05, **P < 0.01. The results are graphed as the median (min-max, horizontal line indicates the median) of at least four independent donors in each assay.

We have analyzed the expression pattern of co-stimulatory markers such as CD80 and CD86, and the antigen presentation surface marker (HLA-DR). Our results indicate a differential expression when infected with one or other viral strain. A significantly higher percentage of CD14^+^CD86^+^ cells were observed after C#1 strain infection, while CD80 did not show significant differences between infected cells. On the other hand, P strain-infected macrophages showed the highest percentage of CD14^+^ HLA-DR^++^ cells revealing a differential expression pattern after infection with C#1 or P strain (Figures 3A,B).

Considering the observed macrophage activation induced by JUNV infection, we next analyzed the level of several cytokines in the supernatants of HMDM at 3 dpi. We found a clear distinctive profile, since higher levels of TNF-α, IL-10, and IL-12 were detected in the supernatants of C#1 strain-infected macrophages, but only IL-6 was significantly increased using the P strain. Interestingly, no difference in IL-1 production was observed compared with mock conditions, suggesting no activation of the inflammasome pathway (Figures 3C–G). The percentage of viable cells does not showed significant differences comparing Mock, C#1 and P (90.2, 88.25, and 83.5, respectively) although small increase in AnnV+ cells were observed with P when compared to C#1 and Mock (Supplementary Information S1).

### JUNV Selectively Skews Macrophage Polarization

**Figure 4:**
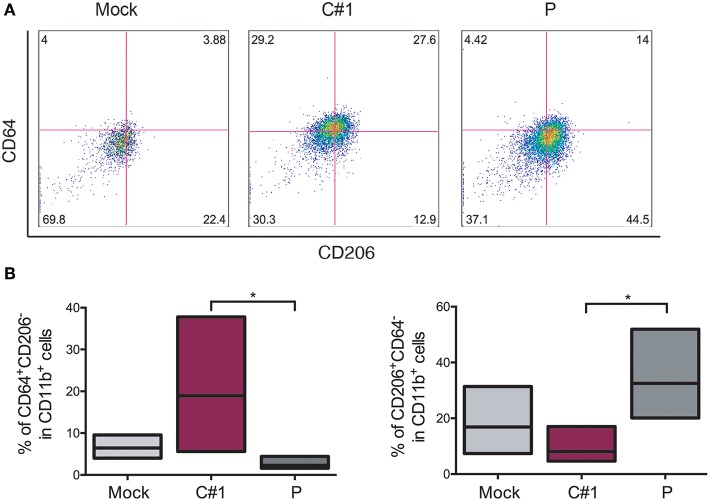
JUNV modulates macrophage M1/M2 polarization. HMDM cells were infected with C#1 or P strains of JUNV (MOI = 1) and at 3 dpi the cells expressing CD64^+^ (M1) and CD206^+^ (M2) on CD11b^+^ were evaluated by flow cytometry. **(A)** Representative dot-plots of CD64 vs. CD206 after gating in CD11b of each condition are shown. **(B)** Independent data are graphed showing a shift to M1 after C#1 infection and to M2 after P infection. Non-parametric One-way ANOVA followed by Dunn's multiple comparison test was used to detect significant differences between groups; *P < 0.05. The results are graphed as the median (min-max, horizontal line indicates the median) of five independent donors. We had set the quadrant threshold based on the FMO of each marker.

Taking into account the fact that JUNV modulates macrophage activation depending on which strain was used, we next evaluated different surface markers to distinguish M1/M2 polarization in HMDM after JUNV infection. The percentage of CD64^+^ (M1), CD206^+^, and CD163^+^ (M2) cells expressed in CD11b^+^ cells were analyzed by flow cytometry. CD11b^+^CD64^+^CD206^−^ cells were increased when cells were infected with C#1 strain as compared to Mock and the P strain with an average of 18 vs. 8% and 3.2%, respectively. However, the M2 phenotype CD11b^+^CD206^+^CD64^−^ was significantly higher after P infection as compared C#1 strain or to Mock with an average of 34 vs. 10.1%, and 17.5%, respectively. This indicates that JUNV modulates polarization according to viral strain pathogenicity (Figures 4A,B). Additionally, another M2 phenotype analyzed as a double positive, CD206^+^/CD163^+^ cells, showed a clear tendency toward an increased percentage after infection with P strain as compared to Mock and C#1 strain infection (44.2 vs. 29.9% and 33.9%, respectively, see Supplementary Information S2).

### The Expression of MERTK Was Differentially Modulated With JUNV Variants

**Figure 5:**
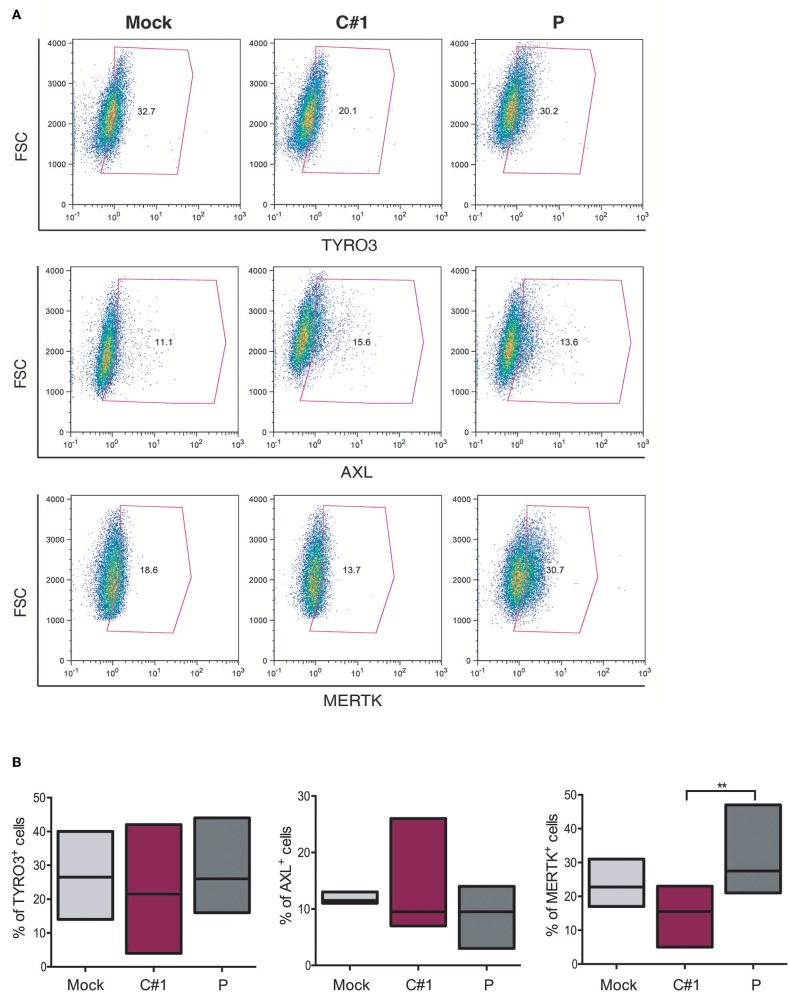
JUNV selectively modulates TAM expression in human macrophages. HMDM cells were infected with C#1 or P strains of JUNV (MOI = 1) and at 3 dpi the expression level of TYRO3, AXL, and MERTK receptors was evaluated by flow cytometry. **(A)** Representative dot plot of each receptor is shown and the percentage of macrophages expressing TYRO3, AXL, and MERTK receptors are graphed in **(B)**. Non-parametric One-way ANOVA followed by Dunn's multiple comparison test was Used to detect significant differences between groups; **P < 0.01. The results are graphed as the median (min-max, horizontal line indicates the median) of seven independent donors. We had set the threshold based on the FMO of each receptor.

The TAM family tyrosine kinase receptors TYRO3, AXL, and MERTK (TAM) receptors have been assigned to have a prominent role in the following: regulating the innate immune response (17); phagocytosis and macrophage polarization by acting in coordination with cytokine signaling (18); and in several aspects of the host response to viral infection (19, 20). Considering our observation that JUNV modulates macrophage polarization and that AXL and MERTK are differentially expressed in pro-inflammatory M1 and anti-inflammatory M2 macrophages, respectively (21), we next evaluated TAM expression in HMDM after infection with both strains. Our results showed that while TYRO3^+^ or AXL^+^ macrophages showed a similar response to infection with the two strains, the percentage of MERTK^+^ cells was down-regulated by C#1 strain and up-regulated by P strain infection, highlighting again a differential macrophage response depending on virus strain (Figures 5A,B).

**Figure 6:**
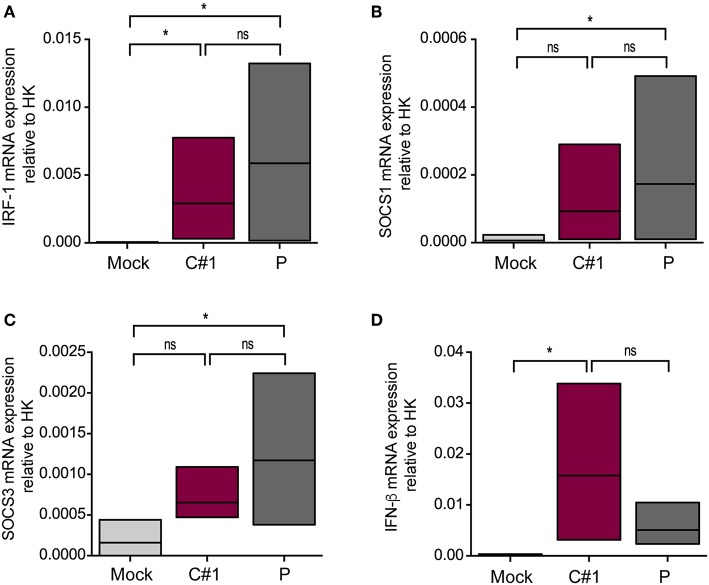
JUNV P enhances transcription levels of IRF-1, IFN-β, SOCS1, and SOCS3. HMDM cells were infected with C#1 or P strains of JUNV (MOI = 1) and after 1 dpi the transcription levels of **(A)** IRF-1, **(B)** SOCS1, **(C)** SOCS3, and **(D)** IFN-β, were studied by RT-qPCR. The Comparative Ct method (2^−ΔCT^) was used to analyze the expression level of the target genes. Non-parametric One-way ANOVA followed by Dunn's multiple comparison test was used to detect significant differences between groups; *P < 0.05. The results are graphed as the median (min-max, horizontal line indicates the median) of five independent donors.

Since activation of MERTK triggers the induction of the suppressor of cytokine signaling 1 (SOCS1) and SOCS3, we next analyzed the transcription level of these genes by RT-qPCR. We also analyzed interferon regulatory factor 1 (IRF-1) as a target gene of infection and a member of the interferon regulatory factor family (22). As expected, we observed higher transcription levels of IRF-1, SOCS1, and SOCS3 concomitant with lower levels of IFN-β in macrophages infected with P strain as compared with mock conditions after 24 hpi (Figures 6A–D).

## Discussion

In the present study, we showed that both attenuated C#1 and pathogenic P JUNV strains induced a phenotypic change in primary human macrophages as early as 1 dpi, that was interpreted as macrophage maturation and/or activation. In addition, we observed similar infectivity titers in the supernatants and a comparable percentage of infected monolayer cells. This stands in contrast with the reported minimal replication of JUNV-XJ (pathogenic) and XJ-Cl3 (attenuated) strains in macrophage cells from adult rats (23), a fact that may be attributed that macrophages were from a different donor species.

Previous studies of human macrophage infection by mammarenavirus have been shown that the non-pathogenic MOPV both replicates and activates macrophages (24) whereas pathogenic LASV replicates, but fails to activate macrophages (25). The lack of activation of LASV-infected macrophages was later associated with sequence differences in viral protein N (26) and involved CXCL10 (27). On the other hand, it has been reported that the non-pathogenic TCRV replicates less efficiently in macrophages than the pathogenic JUNV, but induces a cytokine release not observed in JUNV-infected cells (28). More recently, a differential inhibition of macrophage activation by LCMV and PICV, mediated by the N-terminal domain (NTD) of viral Z protein, has been reported (29). Moreover, LCMV Z NTD leads to increased viral replication and inhibition of IFN responses in macrophages (30), a fact that has been more recently assigned to the Z protein from pathogenic arenaviruses only (31).

Our results partially support to the hypothesis that OW and NW arenaviruses may have different pathogenic mechanisms, at least in macrophages cells (4, 32).

We have previously shown that JUNV infection up-regulates TfR1 in CD34^+^ hematopoietic stem cells (15). Here, we show that both JUNV strains also increased the expression of CD71 in infected HMDM, with P showing higher values. This supports the hypothesis that JUNV promotes its own dissemination not only in undifferentiated hematopoietic cells but also in a differentiated lineage and that P exploits this mechanism more.

The observed differential maturation and activation markers and the cytokine expression profile depending on which JUNV strain infects the macrophages strongly support the notion that C#1 and P strains are able to elicit differential immune responses. In this sense, a higher level of pro-inflammatory cytokines (IL-12 and TNF-α) together with an increased level of a co-stimulatory marker (CD86) demonstrates the ability of the C#1 strain to mount an adequate inflammatory response. This allows the generation of protective immunity against the virus in the absence of disease in the host. By contrast, the pathogenic P strain elicits a more attenuated activation state of macrophages by decreasing the prototypical pro-inflammatory cytokines. However, it also remarkably induces higher levels of IL-6, a cytokine also associated with immunomodulation (33), and increases the percentage of CD14^+^HLA-DR^++^, two signals that indicate an anti-inflammatory response that might allow early immune evasion, facilitating viral dissemination in the host and subsequent disease.

It has been demonstrated that most of the acute viral infections of pathogenic viruses are associated with macrophage activation to a M1 status promoting inflammation (34). Regarding M2, the first studies were carried out with viruses associated with chronic infections, and the first accepted paradigm was that viral infection activates macrophages inducing a M1 profile during the acute phase and an M2 profile emerged during the eventual chronic phase of the disease (34, 35). In many of these studies, the M2-prone response related to an enhanced production of IL-10, which indirectly exerts potent immunosuppressive effects (36–38). Moreover, some viruses, such as herpesviruses and poxviruses, encode functional orthologs of IL-10 (vIL-10s) (39) or IL-6 (33). The viral IL-6 could also inhibit antiviral immunity through inhibition of type I IFN, which allows HHV8 to evade immune detection (40).

Our results clearly show a potent pro-inflammatory response was elicited when macrophages were infected with C#1 strain, concordant with a M1 phenotype. However, the P strain elicited a more anti-inflammatory M2 response, associated with a higher level of IL-6, but not IL-10, an increase in CD11b^+^CD206^+^ and HLA-DR cell expression suggesting that the P strain shifts the macrophage response to a regulatory program (41). In macrophages, IL-10 as well as pro-inflammatory cytokines (TNF-α, IL-12 and IL-6) are produced in response to activation of TLRs 2, 3, 4, 7, and 9, via MyD88 or TRIF, NF-kB, and MAPK pathways (42). The strain P did not stimulate the production of IL-10 neither pro-inflammatory ones such as IL-12 or TNF-α, instead of that, a large amount of IL-6, a cytokine also associated with immunomodulation, was specifically induced by P and not by C#1 strain. Interestingly, in a different model, the IL-6 cytokine has recently been associated with the promotion of the M2 phenotype (43) and described as a potent inducer of SOCS3 (33). Furthermore, the generation of human immunosuppressive myeloid cell populations in human IL-6 transgenic NOG mice has been demonstrated (44).

Very little is known about M2 macrophage polarization during acute viral infection since the early anti-viral response is normally associated with a pro-inflammatory immune response (45). In this regard, a recently transcriptomic analysis of macrophages infected with attenuated or virulent influenza virus strains showed an early and clear profile of genes associated with the M2 phenotype triggered by a pathogenic influenza virus (46).

It has been shown that AXL and MERTK receptors are differentially modulated in cytokine-induced M1 and M2 macrophages, where enhanced levels of MERTK were associated with M2 polarization (21). IRF-1 was initially described as a regulator of type I IFN and MHC-I expression by binding to regulatory regions of their promoters (47). However, IRF-1 is one of the most important IFN-stimulated genes for innate and adaptive antiviral immunity, making a complex network with other transcription factors to finally have a specific response (48–50). In this sense, IRF-2 and 8 can both inhibit IRF-1–mediated induction of transcription competing by promoter binding sites (51, 52), or by blocking protein:protein interactions (53, 54), supporting the hypothesis that viruses can manipulate the induction of IFN and ISGs to enhance their replication. In this sense, the selective modulation of the MERTK, as well as higher levels of IRF-1, SOCS1, and SOCS3 during P strain infection highlight not only the skewing property of this strain, but also its ability to usurp immunomodulatory pathways (TAM/SOCS1 and 3) and potentially use them for immune evasion.

Our results show that JUNV triggers differential macrophage activation and modulates polarization according to viral strain pathogenicity, inducing distinct cell responses that might facilitate correct immune surveillance or viral evasion and dissemination events that end in disease. Thus, the results provide important mechanistic insights into the understanding of JUNV pathogenesis and the multi-faceted host immune responses in arenavirus infection.

Taking our results together with the above-mentioned recent findings by others, it may be speculated that in some acute viral infections, subversion of the conventional M1 pro-inflammatory response to a M2 anti-inflammatory response by acute pathogenic viruses will be more frequent than previously described, and thus is deserving of further study since this may allow the development of potential new candidates for therapeutic targets.

## Data Availability Statement

The datasets generated for this study are available on request to the corresponding authors.

## Author Contributions

MF did most of the experiments. PT performed IF studies. AL and NC made qPCR analysis. AE participated in the generation of macrophages and flow cytometry analysis. VR, JG, and MR edited the manuscript. EC and RG designed the experiments, discussed results, and wrote the manuscript.

### Conflict of Interest

The authors declare that the research was conducted in the absence of any commercial or financial relationships that could be construed as a potential conflict of interest.
