# Supplementary material for: Junin Virus Triggers Macrophage Activation and Modulates Polarization According to Viral Strain Pathogenicity
Source: Front Immunol. 2019 Oct 22;10:2499. doi: 10.3389/fimmu.2019.02499 (PMC6817498; doi:10.3389/fimmu.2019.02499)
Supplement: Supplementary file 1 [file Table_1.docx]

**Table S1**

| Gene name | Primer sequence Forward (Fw) 5´-3 | Primer sequence Reverse (Rv) 5´-3 |
| --- | --- | --- |
| ***EEF1A1*** | TCGGGCAAGTCCACCACTAC | CCAAGACCCAGGCATACTTGA |
| ***IRF1*** | CACTAACATTTCCCCCGAGC | ATGTCCCTGTTCACCCCAAAG |
| ***IFN-B1*** | ATGACCAACAAGTGTCTCCTCC | GGAATCCAAGCAAGTTGTAGCTC |
| ***SOCS-1*** | CACGCACTTCCGCACATTC | TAAGGGCGAAAAAGCAGTTCC |
| ***SOCS-3*** | CTTCGACTGCGTGCTCAA | GTAGGTGGCGAGGGGAAG |
