# Supplementary figures and images for: Junin Virus Triggers Macrophage Activation and Modulates Polarization According to Viral Strain Pathogenicity
Source: Front Immunol. 2019 Oct 22;10:2499. doi: 10.3389/fimmu.2019.02499 (PMC6817498; doi:10.3389/fimmu.2019.02499)

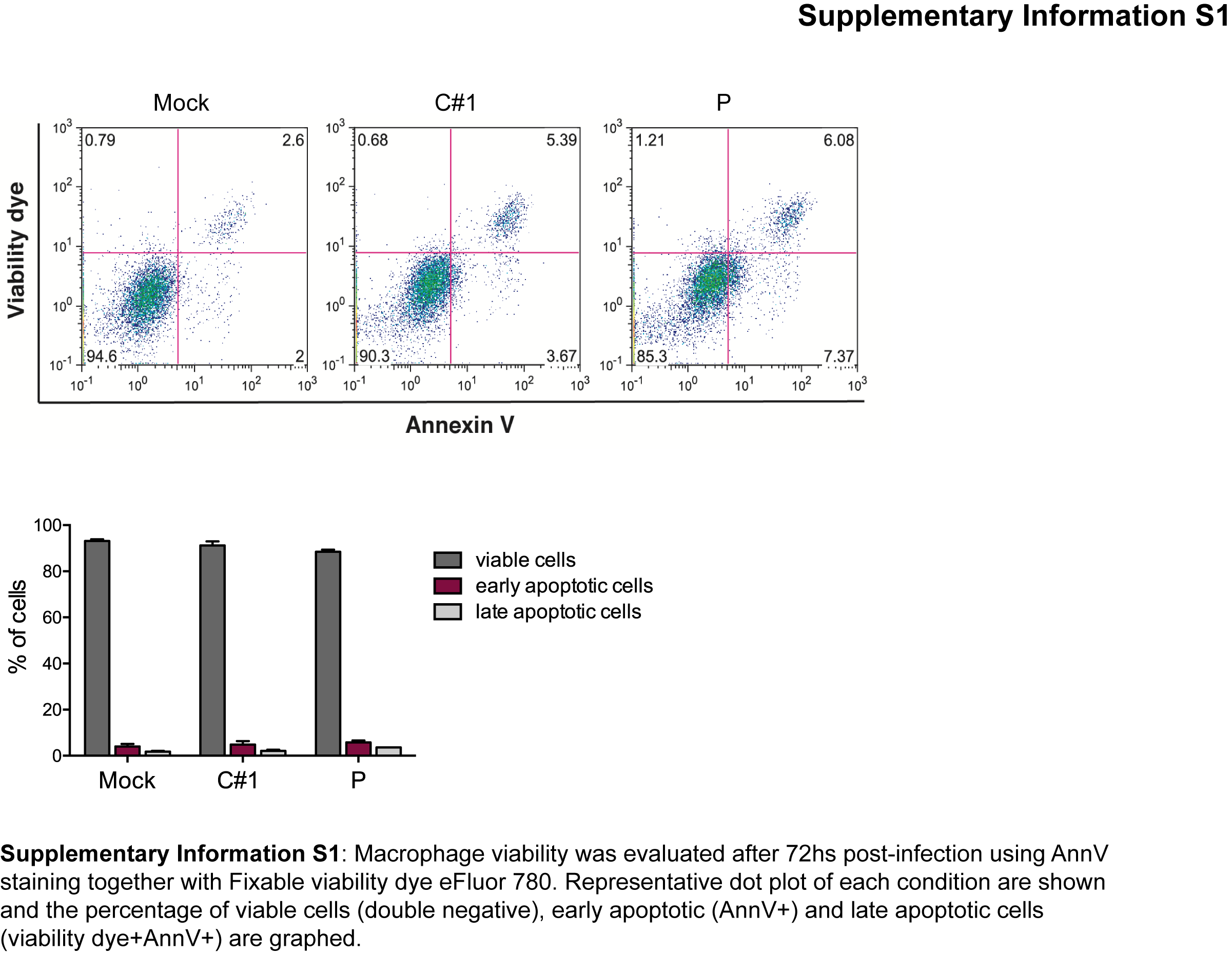

Supplement: Supplementary file 2 [file Image_1.TIF]

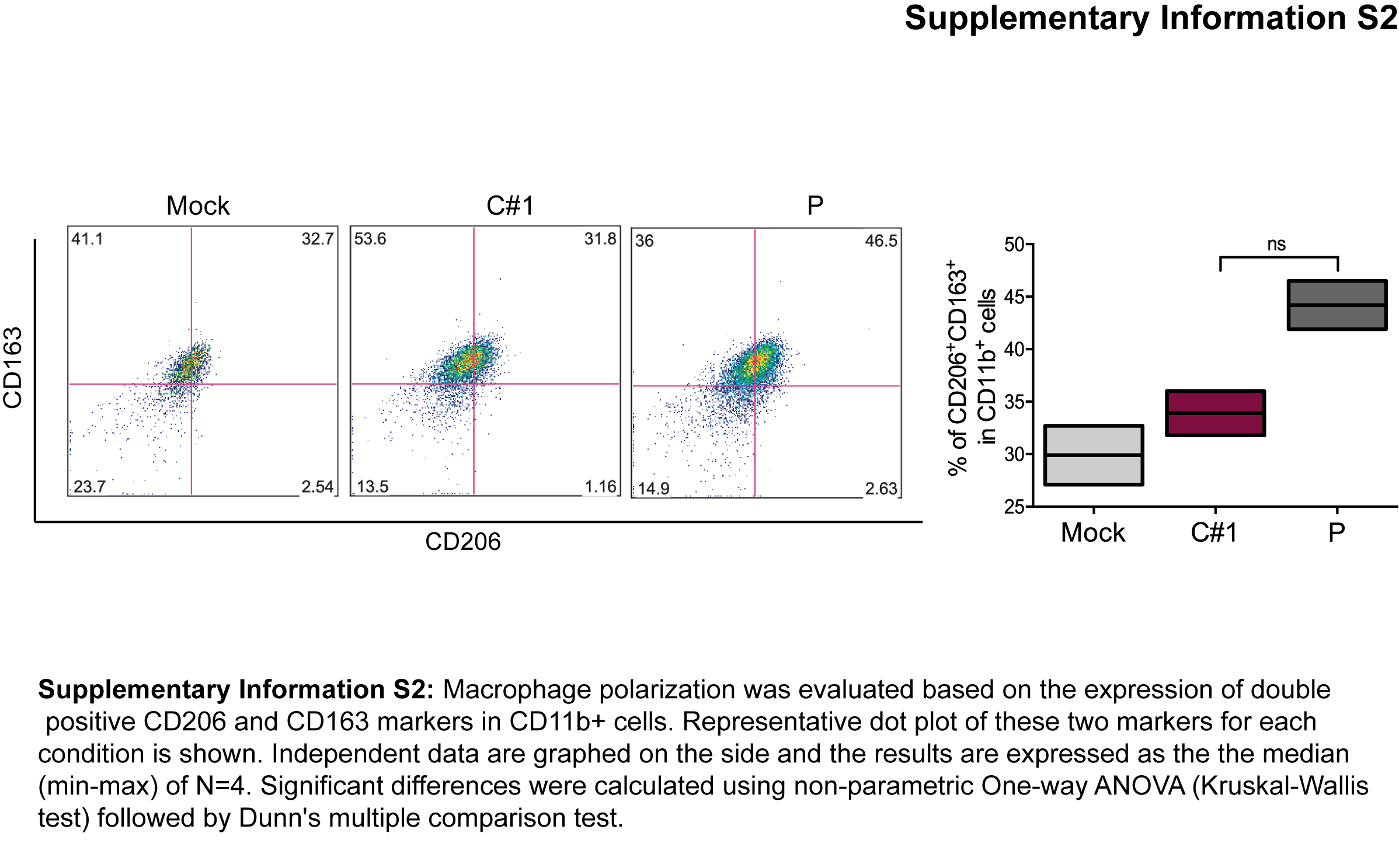

Supplement: Supplementary file 3 [file Image_2.TIF]
